# Érysipèle sur carcinome épidermoïde récidivant de la grande lèvre après radio-chimiothérapie

**DOI:** 10.11604/pamj.2019.33.248.16771

**Published:** 2019-07-23

**Authors:** Younes Barbach, Fatima Zahra Mernissi

**Affiliations:** 1 Service de Dermatologie et Vénérologie, Hôpital Universitaire Hassan II, Fès, Maroc

**Keywords:** Carcinome épidermoïde, radiochimiothérapie, érysipèle, récidivant, Epidermoid carcinoma, radiochimiotherapy, erysipelas, recurrent

## Abstract

The erysipelas is a bacterial infection most frequently due to (85% of cases) streptococcus β-hemolytic of group A (SGA), resulting in non-necrotizing dermo-hypodermitis. Many factors may play a role in its occurrence such as general factors: diabetes, immunosuppression, chemotherapy, radiotherapy as well as locoregional ports of entry such as leg ulcer, an insect bite, intertrigo. The occurrence of erysipelas associated with recurrent squamous cell carcinoma after radiochemotherapy is exceptional, requiring early treatment of dermo-hypodermitis as well as suitable and multidisciplinary management of the carcinoma. The mechanism of the occurrence of erysipelas after radiotherapy coincides with its pathophysiology. Indeed, this therapeutic component, which is very used to treat squamous cell carcinoma, alters lymphatic drainage resulting, very gradually, in lymph fibrosis thus weakening the defense mechanism. Its association with alteration of cellular immunity, also favored by radiotherapy and chemotherapy, can cause the occurrence of erysipelas in these patients. We report the case of a 50-year old female patient with a 1-year history of squamous cell carcinoma of the right labium majus treatment with radiochemotherapy with remission. Eight months later, the patient had recurrence. Evolution was marked by the appearance of a painful hot erythematous plaque on the right thigh suggesting erysipelas, confirmed by laboratory tests which showed an infection. The patient was put on antibiotic therapy with good improvement and then referred to the Department of Oncology for recurrence management.

## Image en médecine

L'érysipèle est une infection bactérienne due le plus souvent dans 85% des cas, à un streptocoque β-hémolytique du groupe A (SGA), donnant un tableau de dermo-hypodermite non nécrosante. De nombreux facteurs peuvent favoriser sa survenue tel que les facteurs généraux: le diabète, l'immunodépression, la chimiothérapie et la radiothérapie, ainsi que les portes d'entrée locorégionales telle qu'un ulcère de jambe, une piqûre d'insecte, un intertrigo. La survenue d'un érysipèle sur un carcinome épidémoide récidivant après radiochimiothérapie est exceptionnelle, cette atteinte nécessite d'abord un traitement urgent de la dermo-hypodermite, puis une prise en charge appropriée et multidisciplinaire du carcinome. Le mécanisme d'apparition de l'érysipèle après radiothérapie rejoint la physiopathologie. En effet, ce volet thérapeutique très utilisé dans le traitement des carcinomes épidermoides altère le retour des vaisseaux lymphatiques et entraine très progressivement une fibrose de la lymphe diminuant ainsi le mécanisme de défense. L'association d'une altération de l'immunité cellulaire, favorisée aussi bien par la radiothérapie que la chimiothérapie faciliterait l'apparition de l'érysipèle sur ce terrain. Nous rapportons le cas d'une patiente âgée de 50 ans, suivie depuis 1 an pour carcinome épidermoide de la grande lèvre droite, traitée par radio-chimiothérapie, avec rémission. Huit mois plus tard, la patiente présentait une récidive de son carcinome, l'évolution était marquée par l'apparition d'un placard érythémateux chaud douloureux de la cuisse droite évoquant un érysipèle, confirmé par le bilan biologique infectieux, elle a été mise sous antibiothérapie avec bonne amélioration puis adressée en oncologie pour la prise en charge de sa récidive.

**Figure 1:**
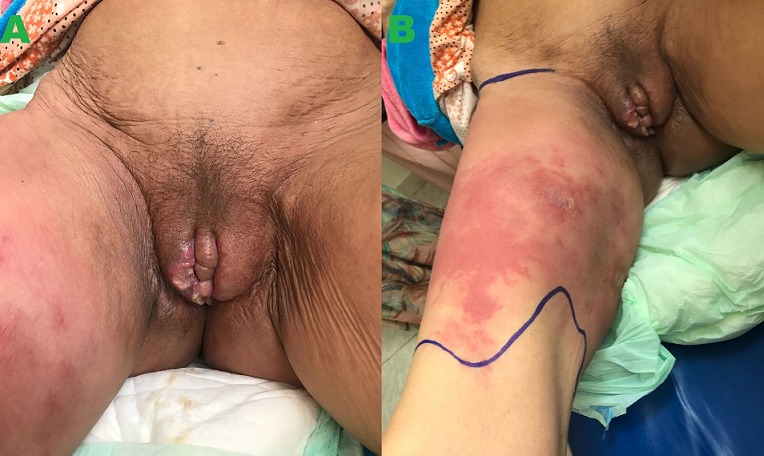
Placard érythémateux chaud douloureux bien limité de la cuisse droite sur tumeur de la grande lèvre droite

